# Socializing Sensorimotor Contingencies

**DOI:** 10.3389/fnhum.2021.624610

**Published:** 2021-09-15

**Authors:** Annika Lübbert, Florian Göschl, Hanna Krause, Till R. Schneider, Alexander Maye, Andreas K. Engel

**Affiliations:** ^1^Department of Neurophysiology and Pathophysiology, University Medical Center Hamburg-Eppendorf, Hamburg, Germany; ^2^Department of Psychiatry and Psychotherapy, University Medical Center Hamburg-Eppendorf, Hamburg, Germany

**Keywords:** sensorimotor contingencies, coupling, prediction, human–robot interaction, coordination dynamics, joint action, autism spectrum disorder

## Abstract

The aim of this review is to highlight the idea of grounding social cognition in sensorimotor interactions shared across agents. We discuss an action-oriented account that emerges from a broader interpretation of the concept of sensorimotor contingencies. We suggest that dynamic informational and sensorimotor coupling across agents can mediate the deployment of action-effect contingencies in social contexts. We propose this concept of *socializing sensorimotor contingencies* (socSMCs) as a shared framework of analysis for processes within and across brains and bodies, and their physical and social environments. In doing so, we integrate insights from different fields, including neuroscience, psychology, and research on human–robot interaction. We review studies on dynamic embodied interaction and highlight empirical findings that suggest an important role of sensorimotor and informational entrainment in social contexts. Furthermore, we discuss links to closely related concepts, such as enactivism, models of coordination dynamics and others, and clarify differences to approaches that focus on mentalizing and high-level cognitive representations. Moreover, we consider conceptual implications of rethinking cognition as social sensorimotor coupling. The insight that social cognitive phenomena like joint attention, mutual trust or empathy rely heavily on the informational and sensorimotor coupling between agents may provide novel remedies for people with disturbed social cognition and for situations of disturbed social interaction. Furthermore, our proposal has potential applications in the field of human–robot interaction where socSMCs principles might lead to more natural and intuitive interfaces for human users.

## Introduction: Grounding Cognition in Action

In recent years, a ‘pragmatic turn’ has been emerging in the cognitive sciences, i.e., a conceptual move away from the classical representation-centered framework toward a paradigm that emphasizes the close relation between cognition and action (for review, see Engel et al., 2013b, 2016). Although such an action-oriented paradigm has been supported by many proponents over the years (e.g., Varela et al., 1991; Clark, 1997; Noë, 2004), it has only recently begun to show conspicuous impact in the cognitive sciences (see Engel, 2010; Menary, 2010; Engel et al., 2013b; Durt et al., 2017). The basic notion is that cognition should not be conceived as the capacity of compiling world-models, which then provide a detached database for independent thinking, planning, and problem solving (Schilbach et al., 2013). Rather, it is emphasized that cognitive processes are so closely intertwined with a body in action that cognition is best understood as enactive, as a form of situated practice rather than disembodied mentalizing (Varela et al., 1991; Noë, 2004; Engel, 2010). Cognition, in this view, is grounded in a pre-rational being-in-the-world based on sensorimotor skills for real-life situations, and core aspects of cognition, such as sensing, perceiving or understanding, become inseparable from doing (Varela et al., 1991; Clark, 1997; O’Regan and Noë, 2001; Noë, 2004). This agrees with phenomenological claims about intricate links between our different senses and the body’s role in thinking (Merleau-Ponty, 1962, 1963), modern anthropological studies of the process of knowledge-making (Myers and Dumit, 2011; Myers, 2015) and recent calls to look beyond analytic ways of knowing (De Jaegher, 2019). Inspired by pragmatist and phenomenological traditions, numerous recent authors have explored the implications of defining cognition as embodied action (Varela et al., 1991; Clark, 1997; Noë, 2004; Pfeifer and Bongard, 2006; Engel, 2010; Menary, 2010; Sheets-Johnstone, 2011; Engel et al., 2013b).

Immediate precursor to the concept proposed in this article, the ‘sensorimotor contingency theory’ (SMCT) by O’Regan and Noë (2001) centers on the notion that perception and cognition can only be understood by considering their inherent action-relatedness. In this framework, sensorimotor contingencies (SMCs) are defined as acquired law-like relations between movements and associated changes in sensory inputs that are continuously probed and refined as we orient in the world (O’Regan and Noë, 2001). The formation of SMCs shows to be highly relevant in cognition (O’Regan and Noë, 2001; Engel et al., 2013b; Maye and Engel, 2013). SMCs are acquired through the agent’s actions, and are deemed constitutive for perceptual processes. For instance, according to the SMCT seeing cannot be understood as computation on internal visual representations. Rather, seeing corresponds to engagement in visual exploratory activity, and consists in sets of skills that are mediated by knowledge in the form of SMCs. This active nature of perception has been emphasized by other approaches as well. However, the concept of SMCT is more radical: it considers action a necessary prerequisite for perception, not just as an output capacity that supports, or interacts with, perceptual processing. Of note, this account does not postulate a unidirectional impact of motor systems on perception but, rather, is compatible with the notion of dynamic sensorimotor interactions in reentrant processing loops (Engel, 2010). There is increasing evidence from work in neuroscience, psychology and robotics supporting the SMCT perspective (e.g., Frith et al., 2000; Maravita and Iriki, 2004; Gallese and Lakoff, 2005; Schubotz, 2007). For instance, neuronal response properties in sensory brain regions strongly depend on action context (Gallant et al., 1998), perceptual scene segmentation is facilitated by the active use of the objects (Bergström et al., 2011), and processes like attention and decision-making have been shown to be strongly related to activity of motor regions (Moore et al., 2003; Donner et al., 2009). Thus, SMCs have been proposed as a framework to define object concepts and action plans, suggesting that the mastery of sensorimotor contingencies facilitates goal-oriented behavior (Maye and Engel, 2011, 2012; Engel et al., 2013b; Högman et al., 2013). This implies that SMCs can be relevant over variable time scales beyond the correlation between movements and the immediate changes in sensory inputs, which are the focus of the original SMCT (O’Regan and Noë, 2001).

In keeping with this pragmatic turn, the concept discussed here suggests an action-oriented framework for social cognition in biological and artificial agents. Our proposal is to ground even complex modes of social interaction in the continuous dynamic coupling between agents and their environments. Successful social interaction, thus, does not come about exclusively through the theories that a detached observer holds about the intentions, beliefs and personalities of other agents (Carruthers and Smith, 1996) but – as we will argue – to a substantial extent via the formation and management of shared rhythms and patterns at the level of embodied sensorimotor dynamics. As will be discussed in greater detail below, our proposal is related to and inspired by other action-oriented concepts of social cognition that have emphasized the relevance of coordination dynamics (Tognoli and Kelso, 2014), of socially salient movement patterns (Lindblom and Ziemke, 2006), motor mimicry (Wang and Hamilton, 2012) and joint embodied action (Sebanz et al., 2006). Notably, earlier proponents of an enactive view of social cognition have suggested that even complex types of social interactions may be grounded in basic sensorimotor patterns that enable the dynamic coupling of agents (De Jaegher et al., 2010, 2017). Supporting this view, evidence is available that interactive sensorimotor dynamics provide substantial clues to social understanding (Di Paolo and De Jaegher, 2012), give rise to high-level processes such as shared intentionality (Sebanz et al., 2006) and empathy (De Waal and Preston, 2017), and are highly relevant for interpersonal affiliation, trust and prosocial behavior (Keller et al., 2014).

In the concept proposed here, the notion of SMCs is substantially broadened beyond its original scope (O’Regan and Noë, 2001) to include the learning and deployment of action-effect predictions on longer time-scales and more complex levels of processing. Previously, we have suggested that SMCs can be deployed, for instance, to acquire object concepts and to achieve prediction and action planning (Maye and Engel, 2011, 2013). Here, we propose that the relevance of SMCs is not limited to cognitive processing of the individual, but extends into the effective interactions between agents in social context. Since in our view these socially shared contingencies are constitutive for social cognition, the influence of others cannot be discarded when seeking to explain individual cognition or behavior: individual and collective processes become irreducibly linked. In the following, we use ‘socializing sensorimotor contingencies (socSMCs)’ as a shorthand for the proposal to ground the development and instantiation of social cognition in shared action-effect contingencies.

## Unpacking the socSMCs Concept

The socSMCs concept moves away from the classical notion that presumptive higher levels of cognition (e.g., self-recognition, perspective-taking, planning, complex reasoning) might differ fundamentally from presumed basic levels of sensorimotor processing (such as perception, multisensory integration, or motor coordination). This aligns well with the notion that both domains of cognition rely on common neural architectures and computational principles (Keller and Mrsic-Flogel, 2018), and evidence that brain regions embodying complex cognitive functions do not differ in principle from modules involved in more basic functions (Douglas and Martin, 2004). Where classical cognitivism might ask, ‘How would we understand the world, other than by generating models about it?,’ the socSMCs concept acknowledges the role of abstract reasoning, but puts equal emphasis on collective sense-making processes that arise only in relation to our physical and social environments. Thus, the socSMCs concept suggests in principle shared neural mechanisms for all our ways of engaging with our environment, and views structures and activities outside of our central nervous system as essential for our cognitive abilities (Clark and Chalmers, 1998).

A key assumption in the concept of socSMCs is that agents deploy learned action-effect contingencies in social contexts to anticipate outcomes of their own and others’ actions (Brown and Brüne, 2012): I am the initiator of change in the (social) world, and change in the world can be directed at me. Such action-effect contingencies closely relate to the more basic framework of SMCs described above where, e.g., stable perception of the world comes about because we actively learn patterns of correlations between our actions (eye movements) and the ensuing effects (changes in the retinal inputs). We propose that agents’ ability to anticipate and coordinate with others at linguistic and abstract levels may derive from their learning of motivated and embodied action in the world. In other words: how we orient in social contexts is very much an extension of how our body orients in the world. This includes social entrainment, defined by the sensorimotor or informational coupling between agents, and social engagement, i.e., the experience of connectedness or relatedness to other agents. The socSMCs concept predicts that both are grounded in the acquisition and deployment of action-effect contingencies. Further, we assume that both the experience of social engagement and our participation in social entrainment are situated within particular physiological, cultural and environmental contexts, within which they emerge and onto which they also feed back.

**FIGURE 1:**
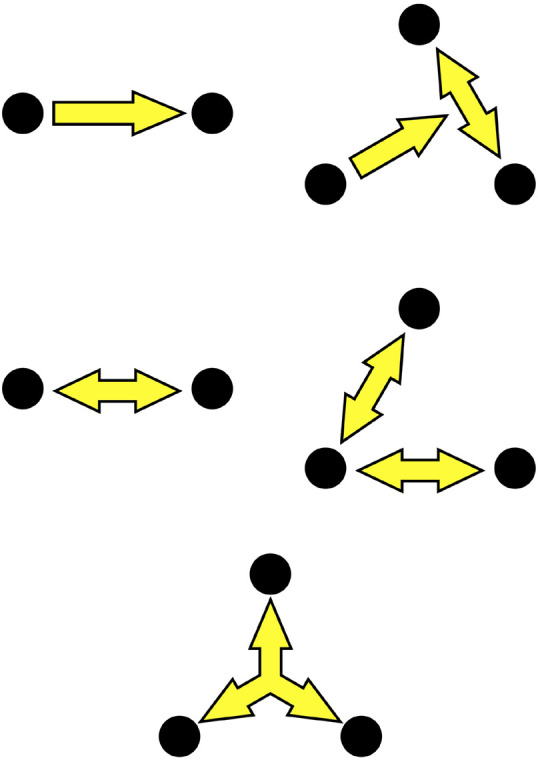
Three hypothesized levels of SMCs in social interaction: **(Top)** Check SMCs may be mediated by unidirectional coupling between two agents (left) or from one person to other interacting agents (right). **(Middle)** Sync SMCs involve reciprocal coupling between two or more agents. **(Bottom)** Unite SMCs are conceived as emergent higher-order correlation patterns in the group dynamics.

Another central assumption in the socSMCs concept is that social interaction can best be conceptualized in terms of dynamic coupling at different scales (Hasson et al., 2012; Engel et al., 2013a; Keller et al., 2014; Hasson and Frith, 2016; Kelso, 2019). We propose to differentiate three levels of complexity of social coupling, reflecting different stages across which interactions are established in a multi-agent system (Figure 1). We term these ‘check SMCs,’ ‘sync SMCs,’ and ‘unite SMCs,’ respectively, to denote that they may correspond to distinct stages, or modes, of social entrainment. These levels are best conceived as points on a continuum, with potential co-occurrence of modes of relating. Across these different levels of socially deployed SMCs, coupling is established over an increasing set of degrees of freedom of the interacting multi-agent system. At the first level, check SMCs involve unidirectional coupling, one agent predicting another agent’s actions or the interaction between several other agents. Behaviorally, this may lead, e.g., to entrainment of one agent to a group of other agents. At the next level, sync SMCs enable bidirectional coupling, with both agents mutually sharing, attending to and predicting each other’s sensorimotor actions. This reciprocity may then lead to genuine interactions and mutual entrainment of behavior, facilitating cooperation, joint attention, turn-taking, and shared action goals. At the third level, we suggest unite SMCs as a hypothetical coupling mode that may promote group-related, multidirectional coupling. Unite SMCs might be characterized by the emergence of interaction patterns that cannot fully be explained by the pairwise interactions among the group members, and attain a certain amount of autonomy over them (see also De Jaegher et al., 2017). For brain networks, there is evidence to suggest the occurrence of such higher-order coupling modes. Thus, it has been shown that cortical activity contains correlation patterns involving spikes from three or four neurons more often than predicted from pairwise correlations, and that such higher-order patterns relate to information encoding and behavior (Montani et al., 2009; Shimazaki et al., 2012). We hypothesize that similar higher-order dynamics might occur for social coupling modes. Such group dynamics may play a key role in group mental states, shared habits, and group affect. At this level, the emergent macroscopic pattern of multi-agent coupling may be stable enough to provide a new source of entrainment for individual agents, beyond the impact of pairwise interactions, as has been observed, e.g., in studies on collective dance improvisation (Himberg et al., 2018).

**FIGURE 2:**
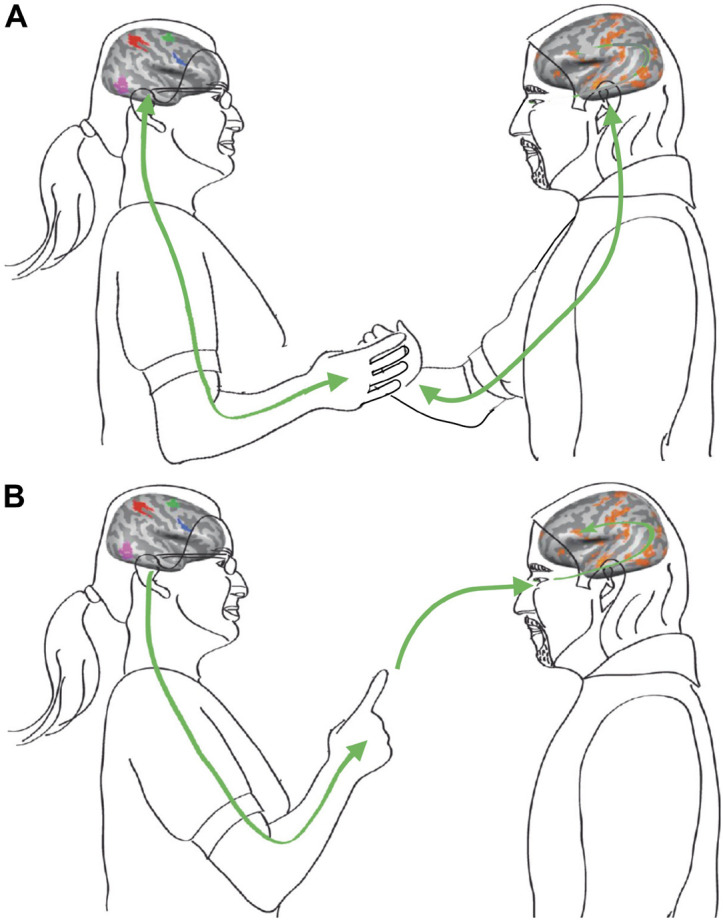
Social interactions may involve proximal and distal types of SMCs. **(A)** Proximal sensorimotor coupling through direct physical contact, involving haptic sensing and kinesthesia. **(B)** Distal sensorimotor coupling based on distance senses including vision and audition to feed action-perception loops. Modified from Hasson et al. (2012).

We suggest that these types of SMCs may take effect over different temporal and spatial ranges, depending on the setting and the mechanisms involved in the interaction. In this context, it may be useful to distinguish between ‘proximal’ and ‘distal’ interactions (Figure 2; cf. Pezzulo et al., 2019). While proximal interactions involve direct physical contact and sensorimotor coupling, distal interactions promote social entrainment by information flow between agents without direct physical coupling. Both proximal and distal social coupling abound in everyday life. Real-world scenarios involving proximal interactions with direct sensorimotor coupling include, for instance: greeting habits, like a handshake or a hug, where mutual dynamic entrainment is highly relevant for signaling the quality of a social relation; joint lifting or carrying of heavy objects that cannot be handled by one person alone, e.g., when moving a household; or dancing together as a couple, where sensorimotor coupling creates the synergy and togetherness enjoyed by the dancers. Examples for distal SMCs in social context include: social mimicry, i.e., an involuntary tendency to imitate or synchronize with postures and gestures of a conversation partner; team sports, ranging from synchronized swimming to coordinated group dynamics in volleyball or soccer; performance of musical ensembles engaged in joint improvisation, or the informational coupling between conductor and orchestra through embodied movement cues. Of note, distal interactions based solely on informational coupling can also take effect in fully virtual settings such as, e.g., in online gaming or in a video conference, provided that the agents can engage in meaningful action-effect contingencies.

The socSMCs concept treats individuals engaged in an interaction as one system. It therefore requires methods suited for the analysis of complex systems, since they may best capture the reciprocal adaptation that underlies coordination and communication (Fusaroli and Tylén, 2016; Gallotti et al., 2016). To this end, we suggest that measures used to quantify coupling within brains (for review, see Engel et al., 2013a) could prove equally useful to quantify the degree of coupling between individuals and their environment. Dynamic functional coupling is considered a key feature of neuronal activity, which exhibits rich spatiotemporal patterning and strongly modulates cognitive processing. Measures used to quantify functional coupling in the brain include coherence, power envelope correlation, information-theoretic measures or multivariate autoregressive models (see, e.g., Engel et al., 2013a; Hutchison et al., 2013; Bastos and Schoffelen, 2015). Much of this coupling is intrinsically generated, that is, not imposed by entrainment to an external stimulus or movement, but emerging from the connections within neuronal networks. There is clear evidence for two distinct types of coupling modes, which seem to be based on different coupling mechanisms (Siegel et al., 2012; Engel et al., 2013a). One type arises from phase coupling of band-limited oscillatory activity, whereas the other results from coupled aperiodic fluctuations of power envelopes. These two coupling modes (phase coupling vs. envelope coupling) differ in their dynamics, their spatial distribution, the time scales over which they operate and they likely support different functions (Engel et al., 2013a). Envelope coupling might reflect co-activation of regions on slower time scales and, thus, might facilitate the participation of brain areas in an upcoming task. Phase coupling, in contrast, represents coupling on faster time scales which presumably generates highly specific dynamic links within networks defined by envelope coupling. As part of the socSMCs concept, we propose that these intrinsic coupling modes are complemented by extrinsic coupling modes, i.e., coupling patterns that reflect the interaction of the brain with the body and its environment, including the social context (Figure 2; cf. Hasson et al., 2012; Hasson and Frith, 2016; Pezzulo et al., 2019). We propose that such extrinsic coupling modes may play a key role in enabling coordinated interaction of multiple brain systems with both body and environment, and that they may be particularly relevant for interaction with the social world. These extrinsic coupling modes should not only become evident at the level of behaviors or movement kinematics, but also give rise to inter-brain coupling in settings where neural signals can be concurrently recorded from two or more subjects (see section on ‘Extrinsic neural coupling modes’ below).

In summary, we suggest the notion of coupling with varying levels of complexity (check, sync and unite SMCs) and an integrated perspective of intrinsic and extrinsic coupling modes to be particularly helpful to understand social behavior. A key prediction is that changes of social entrainment, i.e., proximal or distal sensorimotor coupling, should be associated with changes in social engagement, which may be quantified by subjective ratings of the interaction quality or the degree of cooperation. Thus, we expect that a modulation of social coupling modes, in particular at the level of sync SMCs and unite SMCs, should lead to changes in presumed high-level social cognitive phenomena, such as mutual trust or empathy (Froese et al., 2014; Keller et al., 2014; Llobera et al., 2016). To achieve such a modulation, entrainment through shared perceptual and sensorimotor rhythms is likely to be an important mechanism. Conversely, fluctuations in social engagement might also lead to a differently organized dynamics of intrinsic and extrinsic coupling modes. Thus, for instance, the dynamics of sensorimotor coordination of two individuals should be influenced by social-cognitive factors such as shared intentionality or joint attention. Furthermore, the socSMCs concept emphasizes the continuity between low-level SMCs, which directly involve sensory and motor areas, as well as basal ganglia and cerebellum, and socially deployed action-effect contingencies. Thus, we hypothesize that there may be a strong overlap regarding the brain networks involved in both the former and the latter, as well as an interaction between the intrinsic and extrinsic coupling modes subserving the different types of SMCs. Moreover, with its focus on shared perceptual and sensorimotor rhythms as a core part of the architecture of social cognition, the socSMCs concept leads to the hypothesis that disturbances of these coupling modes may contribute to clinical deficits in social cognition, and that interventions at this level may provide an important tool to promote well-being at an interpersonal level.

## Relation to Other Concepts of Social Interaction

According to the socSMCs concept, social interaction strongly depends on dynamic coupling between agents and their environment, hence a deeper understanding of this interaction dynamics promises to provide important insights into social cognition. Our view shares aspects with the interactionist concept of social cognition (Di Paolo and De Jaegher, 2012; Di Paolo et al., 2018; De Jaegher, 2019) which proposes an extension of the enactivist position to social and affective domains, emphasizing that sense-making occurs in a participatory way and that core aspects of cognition are inherently relational (De Jaegher and Di Paolo, 2007; De Jaegher et al., 2010; see also Durt et al., 2017). The proponents of this enactive view of social cognition emphasize the relevance of self-other contingencies for the coordination between agents in the interaction process (McGann and De Jaegher, 2009). However, a difference to the socSMCs concept is that a relation between social entrainment and intrinsic dynamics of the agents, in particular intrinsic neural coupling modes, is not considered. Furthermore, our concept agrees well with the joint action model by Knoblich and Sebanz (2008), which creates a close link between shared intentionality and joint action, based on the consideration of scenarios with different levels of complexity and flexibility of social interaction. However, the aspect of dynamic coupling is not considered in this model which, rather, focuses on the representation of perceived action in the agents (Sebanz et al., 2006; Knoblich and Sebanz, 2008).

Relations also exist to the concept of ‘coordination dynamics,’ which originated from earlier ideas on self-organizing pattern formation (Tognoli and Kelso, 2014; Tognoli et al., 2020). Coordination dynamics applies dynamical systems theory to biological networks, suggesting that a system is best described by looking at the coupling of its parts via mutual information exchange. An important distinction at the heart of this dynamical view is between (1) coupling of system components with similar dynamics, leading to formation of attractors or multistability; and (2) coupling of system parts with dissimilar dynamics, which prevents phase-locking and leads to metastability, i.e., integrative and segregative tendencies alternate in the interaction dynamics. Tognoli and Kelso (2014) have suggested that these two modes of coupling (multistable vs. metastable) might be useful to describe social coordination. Metastability is particularly interesting also because it represents a state of collective dynamics where new information can be created (Tognoli and Kelso, 2014). The application of this concept to the case of social interaction has been shown to provide very useful tools for the analysis of the interaction dynamics, such as coupled oscillator models (Tognoli et al., 2020). Yet, the focus of this approach has so far been on behavioral aspects of the coordination dynamics and not primarily on the explanation of social cognition and social perception.

Of note, the socSMCs concept differs from classical concepts in social neuroscience. A major focus of work on the neural foundations of social cognition has, in the past decades, been on the capacity of the brain to mirror the actions of others, thus enabling the simulation and representation of other agents’ mental states (Gallese and Goldman, 1998). One of the highly interesting aspects of this approach is its strong emphasis on the role of motor and premotor systems in social cognition. Neuroimaging studies have identified brain areas and networks that are activated during tasks involving mentalizing, empathy or mirroring (Stanley and Adolphs, 2013). A relation between motor control and social cognition is also suggested by work on motor mimicry, an unconscious and spontaneous form of interpersonal coordination, which is likely mediated by the mirror neuron system (Wang and Hamilton, 2012). Along the same lines, De Waal and Preston have proposed a perception-action model of empathy, which postulates the emergence of empathy from basic sensorimotor processes and overlapping representations for performing and observing actions (De Waal and Preston, 2017). Several approaches have suggested a key role for predictive mechanisms in social cognition and also have explored their relevance for disturbed social processing (Blakemore and Decety, 2001; Brown and Brüne, 2012; Sinha et al., 2014). Sokolov et al. (2017) and Sokolov (2018) have highlighted the potential relevance of cerebellar circuits for signaling of prediction errors in social context. In contrast to the majority of the concepts that have been developed in social neuroscience so far, the socSMCs concept focuses on low-level sensorimotor interactions leading to social entrainment and engagement and, vice versa, the influence of social context on the development of basic sensorimotor relations. Pezzulo et al. (2019) emphasize the role of sensorimotor communication in social interaction scenarios of different complexity but without any link to the concept of sensorimotor contingencies. Hasson et al. (2012) and Hasson and Frith (2016) have proposed that social interactions involve the informational coupling of the perceptual system of one brain to the motor system of another which can lead to behavioral alignment, e.g., in verbal communication. However, these authors do not explicitly consider the link between such an extrinsic coupling to intrinsic coupling modes.

The socSMCs concept also differs from classical concepts in social cognition research, in particular, from theory of mind-based approaches. The concept of a theory of mind refers to the idea that a person is aware of the existence of their own subjective experience of the world, and the difference to that of another person. As such, research into this direction describes and promotes social interaction as mediated by theory-theory or simulation-theory (Carruthers and Smith, 1996; Gallese and Goldman, 1998), both of which invoke a meta-level of social cognition, and a distancing from the ongoing moment-to-moment interaction with other agents. In contrast, the socSMCs concept emphasizes the role of more basic and immediate processes of social sense-making, seeking to explain how abstract or higher level insights and decisions come about and are informed by bodily, dynamic and situational factors. This notion also aligns well with evidence from developmental research, suggesting that early in development, the social interaction modes emphasized in the socSMCs concept have primacy and are required to ground other, more explicit modes of social cognition (Campos et al., 2000; Di Paolo and De Jaegher, 2012). Rather than foregrounding models that we hold about others and our interactions with them, the socSMCs concept promotes a picture in which agents co-create shared effects in the world and, thus, understand sociality through the experience of enacting ‘we-modes’ (Varela et al., 1991; De Jaegher and Di Paolo, 2007; De Jaegher et al., 2017). It should be noted that both ways of knowing matter: cognitive model-based prediction and dynamic social coupling, both involve habitual as well as creative components, mutually influence one another and contribute to our flexible engagement with the world (see also Pezzulo et al., 2019). Nonetheless, given the frequent lack of intra- and interpersonal sensorimotor, and experientially lived aspects of cognition in representational approaches, the socSMCs concept is an invitation to keep abstract reasoning and embodied relating at par, acknowledging that the two ways of understanding rely on each other.

## Social Coordination Dynamics

A major implication of the socSMCs concept is a shift in terms of what should be considered as core mechanisms of social cognition. How do we come to understand each other, work on a task together, or settle a dispute? According to the concept advocated here, for multiple agents to act together and understand one another, they must first and foremost find a way to coordinate their sensorimotor engagement with the world and with one another.

**FIGURE 3:**
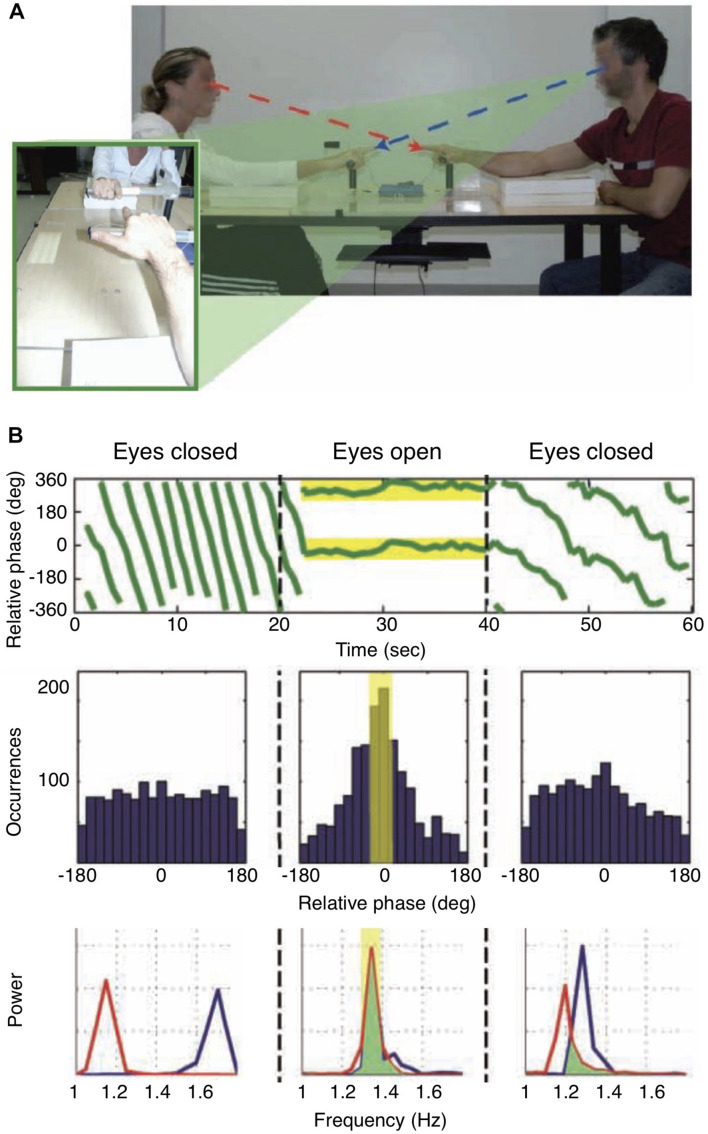
Coordination dynamics in social interaction. **(A)** Experimental setup. Participants were seated opposite to each other and instructed to move their index finger up and down continuously, either with eyes open or eyes closed in separate periods. Importantly, no specific instructions about the coordination of the finger movements were given. **(B)** (Top) Relative phase of the finger movements, indicating synchrony when participants had their eyes open and were viewing each other’s movements. (Middle) Occurrence of relative phase lags of movements. With eyes open, zero phase lag dominated the distribution. (Bottom) With eyes open, participants adopted the same movement frequency; of note, movement frequencies remained similar when participants closed their eyes again. Modified from Oullier et al. (2008).

The importance of sensorimotor coordination for joint action is particularly evident in behaviors involving shared rhythms such as the applause of an audience which can occur in spontaneously emerging synchrony across many individuals. The dynamics of social coordination has been studied, for example, during rhythmic finger movements carried out by dyads of participants with and without visual feedback regarding their own and the other’s movements (Oullier et al., 2008; Figure 3). In epochs with visual feedback, phase synchrony emerged spontaneously between the finger movements, although the participants had not received any particular instruction about how to relate to the partner’s finger movements. Of note, the effect of social entrainment persisted after periods of phase synchronization when visual feedback was eliminated by closing the eyes (Figure 3). This study provides a typical example for what we have termed sync SMCs above (Figure 1). The authors conclude that general features of coordination dynamics, such as multistability and phase transitions, which are observed in a broad variety of self-organizing dynamical systems, are also highly relevant in social interaction. These conclusions are also supported by recent work on joint rushing, i.e., the unconscious increase in pace that can occur during synchronized rhythmic activities (Wolf et al., 2019).

Further prime examples for social entrainment are provided by the coordination dynamics among musicians during ensemble performance (reviewed by Keller et al., 2014). In contrast to more basic laboratory paradigms, entrainment in musical ensembles requires coordination of complex movement sequences with variable temporal patterning. It has been suggested that several cognitive and sensorimotor capacities are required for successful social coupling in such complex settings, including (i) temporal adaptation, supported by mechanisms such as phase correction and period correction; (ii) attention to both the results of own actions, actions of the partners and the joint ensemble output; and (iii) anticipation of action outcomes based on highly precise temporal prediction capabilities (van der Steen and Keller, 2013; Keller et al., 2014). These studies in musical ensembles provide evidence for an impact of sensorimotor coordination on social cohesion, cooperation and trust and, overall, they provide a highly relevant case where synchronous group entrainment can enhance social affiliation (D’Ausilio et al., 2015). Similar conclusions have been reached in the study of musical improvisation involving duets or larger ensembles (Walton et al., 2018). Seeking to understand how musicians communicate and engage socially in an under-determined performance context, Walton et al. (2018) ascribe a central role to shared temporal structure that provides the foundation for performers to interpret and respond to the acts of their partners. Such shared rhythms may provide the basis for what we have termed unite SMCs and for more complex forms of social expression.

It should be emphasized that coordination dynamics is, of course, also relevant in non-rhythmic behaviors. Joint attention may serve as an example here (Sebanz et al., 2006). Joint attention is an important feature of social interaction, consisting in the capability of several agents to simultaneously direct their attention toward the same object. The capacity for engaging in joint attention is frequently taken to indicate the deployment of theory of mind in the participating agents. However, the prominence of sensorimotor components in establishing and sustaining episodes of joint attention, e.g., eye and head movements, pointing and vocalizations, suggests that the concept of socSMCs may be well-placed to account for important parts of joint attention without the need to invoke theory of mind abilities (Maye et al., 2017). For example, exchanging looks or alternating gaze direction between the partner and the object of interest is a simple but powerful mechanism that can establish the mutual awareness of being jointly engaged in a perceptual episode. In addition to gaze perception, head and body orientation may be used as well to infer the target of attention. This view receives support from behavioral studies in humans showing that providing the partners with information about each other’s gaze can significantly enhance performance in a collaborative search task (Wahn et al., 2015). The socSMCs concept refutes the necessity of explicitly detecting and representing the attentional state of an interaction partner. Rather, it highlights the efficacy of the co-attender in modulating the interaction between both partners and between them and the attended object. This transforms the problem of detecting a state into one of establishing a coupling. Jointly attending agents are then organized through this coupling, offering them opportunity windows of coordinated engagement (Fantasia et al., 2014). Common foci of attention are not just passively shared; rather, the co-attenders also shape them, extend them over time by embedding them in task contexts and conventionalize them in terms of canonical forms in the culture (Bruner, 1995).

Similar conclusions are suggested by developmental studies on joint attention. Humans engage in reciprocal attention from as early on as their first hour of life (Trevarthen, 2005; Reddy, 2008; Reddy and Uithol, 2016). Studying vocalizations, movement and gaze of infants interacting with their caregivers, key findings from this field of research include that infants easily follow others’ gaze with their own (Hood et al., 1998; Moore and Corkum, 1998), respond meaningfully even to actions they themselves cannot produce (i.e., their capacities go beyond spectatorial mirroring) and joyfully enter into mutual responding with others, with whom they co-create rhythms and narratives. These developmental steps provide examples for the acquisition of what we term check SMCs and sync SMCs (Figure 1). We grow up in a field of social relations that offer opportunities to participate in joint attention settings, leading us to acquire a know-how about others as bearers of intentions (Reddy, 2003; De Jaegher et al., 2010). Thus, joint attention may be seen as an example for how sensorimotor coupling can lead to an alignment of the agents at the perceptual-motor level as a basic mechanism that contributes to mental alignment in joint action. This may be a seen as preparatory stage for the development of the capability to implicitly take another’s perspective in cooperative situations and later to explicitly understand the other’s perspective as such (Fuchs, 2013). We argue, furthermore, that such basic sensorimotor coordination dynamics influences, adapts and supports our more abstract ability to predict, read and engage with other’s behavior and experience.

Indeed, one of the questions emerging from the socSMC concept is whether subjective feelings of social engagement are associated with motion synchronization between agents, i.e., whether the degree of social engagement can be predicted by the strength of social entrainment. To study this hypothesis one can imagine several scenarios, e.g., situations in which agents synchronize their movements, act together to achieve common goals, play music, or dance together. One study investigating this influence used a three-dimensional mirror game, in which agents had to synchronize their movements (Llobera et al., 2016). Either one of the agents was leading or following, or they jointly improvised without a designated leader and follower. The analysis of motion data and of subjective ratings revealed that the perceived sensation of synchrony could be predicted by parameters of motor synchronization in this mirror game. Especially the speed differences between the agents’ movements were a good predictor for the subjective sensation of synchrony.

Several studies also used objective measures to quantify social engagement, e.g., by the duration of co-confident motion which corresponds to jitter-free, synchronous movements of two interacting agents. Co-confident motion was first described in a one-dimensional version of the mirror-game, a simple joint improvisation task (Noy et al., 2011; Hart et al., 2014; Gueugnon et al., 2016). Here, periods of co-confident motion were associated with increased social engagement and, thus, considered to indicate moments of togetherness. Even physiological parameters such as increased heart rates were shown to be associated with periods of co-confident motion and, moreover, these periods showed correlated heart rates between two improvising agents (Noy et al., 2015). We have recently obtained similar evidence in a joint attention task, in which two agents had to cooperate to determine the motion direction of a visual object on a screen. We observed that autonomic parameters related to heart rate variability could reflect the subjective evaluation of performance in the task (Maye et al., 2020). In other studies, personality traits such as the attachment style (Bowlby, 1969) were used to predict complexity and synchronization of motion in joint improvisation (Feniger-Schaal et al., 2016, 2018).

## Impaired Social Coupling

The concept advocated here also has implications for understanding the basis of social cognition disorders. Impaired communication plays a role in many areas of psychiatric and psychotherapeutic practice, from temporary cases of miscommunication to persistent deviations and impaired social interactions. Communication deficits are a highly relevant aspect in diverse psychiatric disorders, such as schizophrenia and other psychotic disorders (Baltaxe and Simmons, 1995; Fioravanti et al., 2005), depression (Pope et al., 1970) and, in particular, neurodevelopmental disorders of the autism spectrum type (Magiati et al., 2014; Tillmann et al., 2019). The socSMCs concept predicts that patients with social cognitive deficits may suffer from deficits in mechanisms for interpersonal sensorimotor entrainment.

Autism spectrum disorder (ASD) may serve as a specific example for a condition with verbal as well as non-verbal communicative deviations (Lai et al., 2014). First described several decades ago in the context of schizophrenia as autistic thinking (Bleuler, 1911), autism was later investigated by Kanner (1943) and Asperger (1944) and underwent a considerable paradigm shift with the introduction of the autism spectrum (American Psychiatric Association, DSM V). Recently, ASD has been investigated extensively in the fields of psychology, psychiatry as well as clinical neuroscience (Happé and Frith, 2006; Frith and Frith, 2008; Wolfers et al., 2019). With symptoms that range from social and communicative to sensory and motor impairments, ASD’s etiology and pathophysiology are still not fully understood and until today, only very few established treatment options exist.

It has been argued that reduced social entrainment in ASD may relate to impaired perception of affordances provided by other persons’ behaviors (Hellendoorn, 2014). The Gibsonian notion that behavior affords behavior (Gibson, 1986) resonates well with the socSMCs concept proposed here, since it emphasizes the emergence of affordances in joint action and implies a coupling of perception-action loops supporting the social interaction (Hellendoorn, 2014). An immediate application of socSMCs principles to ASD suggests strategies for enhancing social coupling at the sensorimotor level. Brezis et al. (2017), for example, compared autistic and typically developing participants’ behavior on the mirror game, an open-ended task where two players take turns leading, following, and jointly improvising motion using two handles set on parallel tracks. They found that autistic participants had lower rates and shorter duration of co-confident motion, in particular when they were following. These differences remained even when controlling for motor skills. Based on participants’ subjective reports, the authors suggest attention, motivation, and reward-processing as potential mediating factors, and propose to examine the potential of specific training of sensorimotor coordination to enhance patients’ social cognitive abilities. Along these lines, a recent study has investigated the impact of a dance/movement intervention on social cognition in ASD (Koehne et al., 2016). The authors observed that training of movement imitation and synchronization increased emotion inference in adults with ASD.

Another well-studied domain of impaired SMCs in ASD are eye movements. Among the most frequently observed symptoms in ASD, the avoidance of eye contact leads to a range of consequences in social interaction. Studies on human social development show that 2-year-old children with ASD tend to show significantly less visual fixation time on faces, when a video of an actress (acting as a care-giver) was presented (Jones et al., 2008), indicating a very early impairment in a social adaptive behavior that is regarded as evolutionarily vital for survival in humans and shown to be relevant for newborns at very early stages in development (Farroni et al., 2002). This early deficit seems to persist into adulthood, as shown in an eye-tracking study in adults using naturalistic social situations as stimuli (Klin et al., 2002). Importantly, this deficit also causes a lack of active perception in a critical time window in early development, in which basic learning processes drive social and emotional development, and may therefore be closely related to symptoms such as the difficulty to recognize emotional expressions in others (Eack et al., 2015). This difficulty is detrimental to any kind of communication and reported frequently in ASD as one of the most impairing symptoms. The case of gaze aversion exemplifies how active visual perception is intricately linked to both development and learning in social contexts as well as the successful unfolding of communicative acts.

Complementing these behavioral studies, neurophysiological evidence indicates that not only sensory (Robertson and Baron-Cohen, 2017) and motor (LeBarton and Landa, 2019) processing appears deviant in ASD, but also the interplay between these domains. It has been shown in children with ASD that resting state fMRI connectivity is reduced between visual and motor systems (Nebel et al., 2016). The reduction of visual-motor coupling was associated with symptom severity in terms of more severe social deficits. The socSMCs concept implies that social entrainment involves mechanisms for acquiring action-effect contingencies in the social interaction and, thus, a critical role of brain regions involved in prediction of sensory inputs and action outcomes, such as prefrontal cortex, premotor cortex, cingulate cortex, superior and middle temporal gyrus, basal ganglia and the cerebellum (Schubotz, 2007; Brown and Brüne, 2012; van der Steen and Keller, 2013; Sokolov, 2018; Van Overwalle et al., 2019). Accordingly, deficits in such predictive mechanisms should have an impact on social entrainment. Indeed, a key deficit in ASD seems to concern the ability to form flexible predictions, leading to an impairment in processing of new or unexpected sensory inputs (Gomot and Wicker, 2012) and aberrant movement planning in joint action contexts (Gonzalez et al., 2013). Deficits in predictive mechanisms in ASD have also been postulated by Sinha et al. (2014). According to their proposal, an underlying deficit in predictive abilities may account for many of the salient traits in ASD, including sensory hypersensitivities, difficulties to interact with dynamic objects, reduced motor anticipation, as well as difficulties in anticipating the actions of other persons (Sinha et al., 2014). At the neural level, this predictive impairment may relate to alterations in structures involved in prediction like the basal ganglia, anterior cingulate and cerebellum (Sinha et al., 2014; Sokolov et al., 2017; Sokolov, 2018; Van Overwalle et al., 2019). In particular, the cerebellum shows developmental alterations in ASD, including strong expression of ASD susceptibility genes, volume decreases and cellular abnormalities (Wang et al., 2014). This agrees with a role of cerebellar circuits in outcome prediction, signaling of prediction errors and perception of a person’s motion and body language in social context (Sokolov et al., 2017; Sokolov, 2018; Van Overwalle et al., 2019). Deficits in sensorimotor entrainment in ASD have been examined by Wang and Hamilton (2012) and Forbes et al. (2017), who studied motor mimicry in social interaction. They observed that people with ASD can still mimic, i.e., unconsciously copy the actions of others, but do not use social cues like, e.g., gaze to control what to mimic (Forbes et al., 2017). This provides support for the hypothesis proposed here, demonstrating mimicry as a socially relevant coupling mode which influences engagement through sensorimotor entrainment.

## Extrinsic Neural Coupling Modes

To explore the neural mechanisms involved in social interaction, the concurrent observation of brain dynamics ongoing in two (or more) people who communicate, work on a joint task, or improvise together seems highly informative. In recent years, the investigation of inter-brain coupling using so-called hyperscanning methods based on simultaneous electro- or magnetoencephalographic (EEG/MEG) recordings or functional magnetic imaging (fMRI) scans of individuals engaged in a social task has gained attention in social neuroscience (Montague et al., 2002; Schippers et al., 2010; Hasson et al., 2012; Sänger et al., 2013; for a recent review also see Czeszumski et al., 2020). These approaches investigate the neural signatures of dynamic social coordination, the temporal and spatial scales on which brains interact and the correlates of behavioral coordination at the level of brain-to-brain coupling. Hyperscanning paradigms employed to investigate social interactions are manifold, including joint musical performance (Lindenberger et al., 2009; Sänger et al., 2013; Novembre et al., 2016), verbal communication (Liu et al., 2017; Li et al., 2021), decision-making in economic games (King-Casas et al., 2005; Krueger et al., 2007; Jahng et al., 2017; Hu et al., 2018), and sensorimotor coordination and imitation (Hari and Kujala, 2009; Babiloni and Astolfi, 2014; Hari et al., 2015; Liu et al., 2018; Nummenmaa et al., 2018). The intriguing idea of investigating social interactions by simultaneously recording neuronal activity from interacting brains has also been implemented for the investigation of adult-infant interactions (Hasegawa et al., 2016; Leong et al., 2017), pain perception and interpersonal touch (Goldstein et al., 2018), and has been transferred to real-life scenarios such as flight simulations in professional pilots (Toppi et al., 2016) and classroom group dynamics (Dikker et al., 2017).

To identify neural signatures of social interactions, connectivity analyses have been applied to measure both phase as well as envelope brain-to-brain coupling. The quantification of inter-brain coupling in EEG and MEG hyperscanning data includes the assessment of phase-locking between oscillatory activity in specific frequency bands (Lindenberger et al., 2009; Dumas et al., 2010; Sänger et al., 2013), as well as amplitude envelope correlations of oscillatory power (Tognoli et al., 2007; Naeem et al., 2012; Kawasaki et al., 2013). There is growing evidence from EEG/MEG hyperscanning studies that links connectivity between brains to interpersonal coordination and joint action (see for example Dumas et al., 2010; Toppi et al., 2016; Szymanski et al., 2017; Kawasaki et al., 2018; Zamm et al., 2018). Particularly, in experimental paradigms involving rhythmic, musical or motor coordination, the alpha- (or mu rhythm, oscillatory activity ranging from 8 to 13 Hz) and beta- (15–30 Hz) bands seem to mediate inter-brain coupling (Tognoli et al., 2007; Lindenberger et al., 2009; Dumas et al., 2010; Naeem et al., 2012; Novembre et al., 2016; Kawasaki et al., 2018). Besides phase relations, amplitude envelope correlations between brains are computed to investigate slower fluctuations during coordinated behavior (Hari et al., 2015; Zamm et al., 2018), which may be more appropriate considering the timescale of interpersonal sensorimotor coordination.

**FIGURE 4:**
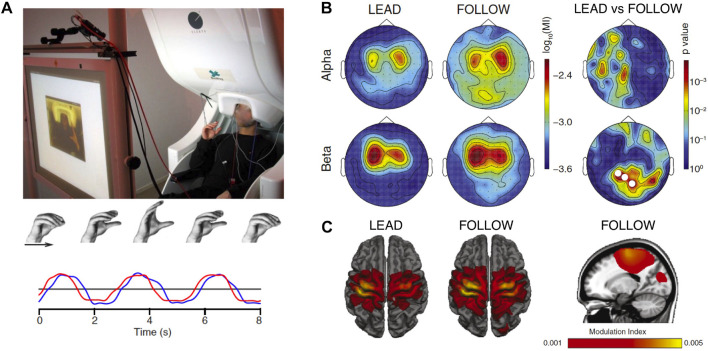
Modulation of brain signals by joint action. **(A)** Experimental setup. Participants were seated in two separate MEG systems and instructed to perform rhythmic precision-grip-like movements in synchrony with their partner, either leading or following the other’s movement. Example movement traces (red, blue) are shown at the bottom, indicating similar movement with slight delay between the participants. **(B)** Modulation of alpha- and beta-band power by the phase of the hand movement in the two conditions. Modulations occurred over central areas and, for beta power, also over visual cortex. Significant differences between the leader and follower conditions (right) occurred only for beta-band power recorded from visual areas. This role-specific modulation of brain activity might be reflecting the need for the follower to coordinate own proprioceptive signals with the visual feedback about the movement of the leading participant. **(C)** Source space projection of the results shown in panel **(B)**. Power modulations are observed in sensorimotor cortex as well as, in the follower condition, in visual cortex. Modified from Zhou et al. (2016).

The socSMCs concept suggests that establishing direct links between movement kinematics and neural data recorded during social interaction might be particularly promising. One way to link neural measurements with movement data in joint action research is exemplified by the work of Zhou et al. (2016). The authors used phase-amplitude coupling to quantify the relation between the phase of hand movement accelerations and oscillatory power in the alpha- and beta-bands during a joint motor task in a dual-MEG setup (Figure 4). The participants had to coordinate rhythmic precision-grip-like movements while brain signals were recorded simultaneously using two MEG systems. The goal of the task was to synchronize the own movements with those of the partner, either leading or following in the interaction. The data show a movement-related modulation of alpha- and beta-band power in sensorimotor cortex and, furthermore, a modulation of beta-band power in visual cortex, which was stronger in the follower compared to the leader condition. The authors suggest that this modulation of oscillatory brain activity might be a signature of the need for the follower to coordinate own proprioceptive signals with the visual information about the movement of the leading participant (Zhou et al., 2016).

Several questions regarding the interpretation of hyperscanning results arise: (i) What is the substrate or underlying mechanism of inter-brain coupling? (ii) How can inter-brain processes shape the experience and behavior of individuals in interaction? (iii) In how far is observation at the brain-to-brain level more informative than, for example, an investigation of interpersonal sensorimotor dynamics? Given that direct coupling between neuronal ensembles of two brains can be ruled out for the lack of neuroanatomical connection, shared or synchronized sensory inputs, and coordinated motor outputs, are potential candidates. In keeping with this idea, Dumas (2011) suggested that when individuals’ perception and action are coordinated, for example in a joint task, inter-brain synchrony may reflect sharing of information via between-individual sensorimotor loops or channels (Hasson and Frith, 2016; Pezzulo et al., 2019). Akin the differentiation of check, sync and unite SMCs, processes favoring the emergence of inter-brain synchrony may be described as ranging from similar external sensory stimulation of both individuals (check), reciprocal interpersonal action (sync), and group behavior that is inspired by a common ground, be it affective, informational or sociocultural (unite). Taken together, available hyperscanning studies provide evidence that sensorimotor or informational coupling between agents can be associated with inter-brain coupling of neural signals, supporting predictions that arise from the socSMCs concept.

Both phase and amplitude coupling methods have been criticized for finding spurious coupling, or hyper-connectivity non-existent in the data (Burgess, 2013; Hari et al., 2015). For example, two neuronal ensembles oscillating at the same frequency show high phase-locking per definition, without necessarily influencing each other. Another criticism observes that the EEG of two individuals taking part in the same experimental protocol likely shows inter-brain synchrony (due to identical sensory stimulation or similar motor output) in spite of a complete absence of interaction (Burgess, 2013; Hari et al., 2015). Circular correlation coefficients, mutual information (Burgess, 2013), or canonical correlation analyses (Campi et al., 2013; Hari et al., 2015; Vidaurre et al., 2019) have been suggested as measures that may avoid such spurious coupling. In addition, comparing inter-brain coupling in real participant pairs with randomly selected pairs (e.g., Bilek et al., 2015; Toppi et al., 2016) might aid the identification of non-trivial synchronization effects linked to the interaction between agents. However, it remains a complex task to differentiate between the diverse communicative processes involved in social interaction and to then identify their respective substrates.

The socSMCs concept argues for an integrative analysis of interaction data, including behavioral coordination in terms of sensorimotor coupling between agents, inter-brain synchronization, and subjectively experienced social engagement. A testable hypothesis is the prediction of self-assessment of social engagement, as measured by questionnaires or rating scales administered during joint action, from measures of behavioral and neural coupling between agents. Supporting this hypothesis, several studies have linked neural synchronization between interacting brains to subjective experience, e.g., feelings of engagement and social closeness (e.g., Dikker et al., 2017) or ratings of pain experience (Goldstein et al., 2018). These findings are complemented by evidence linking movement synchronization to social cohesion and subjective experience (as detailed above and also reviewed in Valencia and Froese, 2020). From the viewpoint of socSMCs, it is desirable to now go a step further and combine measures of social entrainment and social engagement, i.e., sensorimotor coupling, inter-brain synchronization and subjective experience into one model of social interaction.

Hyperscanning setups have also been used for joint neuromodulation of interacting participants, using an interventional approach to further explore underlying mechanisms of inter-brain coupling. In a study involving transcranial alternating current stimulation (tACS) applied simultaneously over motor cortex in two subjects during a joint finger tapping task, movement synchrony was enhanced by in-phase beta-band tACS (Novembre et al., 2017). Another study used dual-brain tACS to augment social interactive learning by enhancing spontaneous movement synchrony (Pan et al., 2020). Future studies might test whether such neuromodulatory interventions that lead to enhanced movement synchrony also have a potential impact on the subjects’ assessment of social engagement.

As discussed earlier, we propose that the socSMCs concept might also provide new angles for neuropsychiatric research and psychological treatment, for example in ASD. Several studies have investigated interpersonally shared sensorimotor rhythms and their role for joint attention, mutual trust or empathy in hyperscanning setups involving ASD patients. These studies have revealed reduced inter-brain coupling in dyads involving ASD participants compared to neurotypical controls, which was associated with the impairment of the social interaction and/or the severity of ASD (Tanabe et al., 2012; Salmi et al., 2013; Hasegawa et al., 2016).

## Relevance for Human–Robot Interaction

We propose that the relevance of sensorimotor entrainment for social coupling not only applies to human social interaction, but can also serve to improve human–robot interaction (HRI). In fact, work in robotics provides early implementations of decentralized embodied executive control (Brooks, 1991). In the development of socSMCs-based robot controllers, the focus lies on algorithms for learning and deploying action-effect contingencies rather than for extracting semantic features from the sensor data, high-level reasoning and action planning and execution as in current mainstream robotics. The socSMCs concept suggests that many of the social action-effect contingencies involved in HRI can be observed by using rather simple features calculated from the sensory data. For example, optical flow can be used to entrain a population of neuronal oscillators by adjusting their phases and frequencies. When a motor control signal is derived from a weighted superposition of the oscillator signals, this model enables a robot to imitate gestures and to synchronize its movements with the human partner (Ansermin et al., 2016). Exploiting the mutual entrainment drastically simplifies the computational complexity of gesture mirroring and achieves millisecond-precision synchronization, which is challenging to accomplish with controllers that require high-level planning processes. Other low-level sensor data, like, for example, from distance sensors, collision detectors or the power consumption of the wheel drive, have been used to learn associations between actions and resulting changes in the sensory input, i.e., SMCs. Basically, sensor readings were combined to form an entry into a memory of SMCs that the robot had explored in the corresponding context. A reward function was used to rank different behavioral options. Together with a history of recently activated SMCs, the robot could develop an understanding of the geometric properties of its environment (Maye and Engel, 2011). This allowed the robot to traverse the space without hitting obstacles not because it was programmed to pull back whenever a distance sensor flagged an imminent collision, but because it inferred from the learned SMCs and its previous action sequence where it was and that moving on would have a detrimental effect.

The reward structure of behavioral options that is conditioned on the recent history of sensorimotor interactions can be conveniently captured by Hidden Markov Models (Maye and Engel, 2013). A powerful feature of this approach is the dual use of the model. Employed as a forward model, imagined or observed sensorimotor sequences can be used to simulate future behavioral trajectories and gauge their outcomes. In the backward direction, histories of sensorimotor interaction can be searched for common patterns which effectively is a way to derive more abstract knowledge from a set of particular interactions that all yielded the same effect.

We hypothesize that implementing social interaction capabilities in a robot which already is driven by knowledge of relevant SMCs may not depend on any critical module or function, as little as social cognition does not require any extra components that a cognitive agent wouldn’t have. Therefore, adapting SMC-based robotic approaches to the social level by including socially relevant, low-level sensorimotor features seems straightforward. A model case for this transition has been made in a study which investigated a scenario where a robot and a human jointly balanced a ball on a plank (Ghadirzadeh et al., 2016). At the first stage, the robot learned the own action-effect contingencies of tilting its end of the plank and the trajectory of the ball. It then collaborated with a human by optimizing the joint goal function which kept the ball on the plank. An example for a real-world scenario that strongly relies on this type of sensorimotor coupling is the joint lifting and carrying of heavy objects, e.g., during removal of furniture to a new home. Reinforcement learning was employed for action selection from learnt SMCs, and residual uncertainty of human actions was modeled by Gaussian processes. The possibility to predict human movements from chunks of past trajectories indicates that human behavior indeed exhibits patterns which can be exploited by robot controllers (Bütepage et al., 2018). Instead of top-down approaches like explicit cost functions or target-specific training data, the authors used a bottom-up, data-driven model that was trained in an unsupervised way. Knowing regularities in the way humans move allows the controller to make predictions about the human’s actions, which greatly limits the space of possible robot movement trajectories and thereby lowers response times (Bütepage et al., 2019). It has to be pointed out that this approach is different from gesture recognition in that it does not attempt to derive abstract descriptions of the movements like pointing or stirring, which is then the basis for decision making and action planning. In the socSMCs framework, the robot is rather controlled by a network of sensorimotor memory traces in which reward-based learning assigned utilities to paths and which can be used by the controller to evaluate behavioral options. More generally speaking, developing HRI on the basis of the socSMCs concept does not suggest to introduce articulated contingency detector modules. Social coordination, rather, results from linking the individual agents’ networks of SMCs through the interaction, thus constituting a global network in which circular causality drives the collective dynamics. Corresponding simulation studies in evolutionary robotics have successfully modeled interaction dynamics in the perceptual crossing paradigm in which participants seek to differentiate a partner, their shadow and a static object – all of which feel the same as you cross them, only two of which move, and only one of which (the partner) responds to one’s presence (Di Paolo et al., 2008).

By making human behavior more accessible for robot controllers, wearable sensors may help bridging the currently very different physical substrates of human and artificial agents and facilitate social entrainment in HRI. For example, data from a head-worn inertial measurement unit can enable a robot controller to learn human movement patterns related to mutual attentiveness, coordination and overall positivity (Hwang et al., 2019). We suggest that HRI feels natural to the extent that SMCs acquired in human-human interaction can be deployed also in the interaction with the robot. This idea has consequences for all aspects of robotic development. For example, synchronized movements, such as when we pass on or carry objects together, require mutual frequency adaptation in the human and the robot. This process runs much more efficient if the intrinsic frequency properties of the human and robotic embodiments are compatible (Ansermin et al., 2017), which can inform the mechanical design of robots, e.g., to size robotic limbs comparable to those of humans. Another effort to narrow the gap between different embodiments and make SMCs acquired in human-human interaction useful in the context of HRI may be the development of methods for endowing robots with facial expressions (Vouloutsi et al., 2019). This may be seen as a gimmick at first; however, from the socSMCs perspective, changing facial expressions support just another subset of SMCs that humans engage in their mutual interaction, which may facilitate also the interaction with the robot.

Thus, socSMCs-based human–robot coupling may enhance computational efficiency through information reduction and yield robot controllers that depend less on abstract explicit internal representations, rendering real-time control of the interaction feasible. A few iterations of the interpersonal sensorimotor loop may activate memories of previous or similar interactions which may then modulate the relative weighting of possible behavioral options that the agents can choose from. This also has the potential to replace rather discrete switching of the active role between the human and the robot with quasi-continuous turn-taking, encouraging the feeling of doing something together as opposed to interacting with a machine.

## Grounding Togetherness in Dynamic Coordination

As pointed out above, the socSMCs concept combines pragmatic (embodied, enactive) approaches with a constitutive role of social interaction, questioning the appropriateness of conceiving minds as independent individual entities (see also De Jaegher and Di Paolo, 2007; Gallagher, 2008; Satne and Roepstorff, 2015; Kyselo, 2016). For the study of human social capabilities, this implies a dissolution of the boundaries between me and the other that pervade classical cognitivist approaches. In particular, the socSMCs concept focuses on the relation between coupling dynamics at neurophysiological and behavioral levels, and the varying degrees of social engagement experienced by the individuals. This is in line with results from studies that used the mirror game, a simple setup in which two players sit opposite each other and coordinate the movement of two handles placed on parallel tracks in front of them. Noy et al. (2011) show that highly jitter-free, co-confident movement goes hand in hand with the highly agreeable experience of togetherness – a subjective merging of self and other, accompanied by the sense that every action is the right one. In a follow-up study, Noy et al. (2015) further showed how both subjective ratings of moving together and objective motion-based markers are predictive of physiological responses like correlated heart rate fluctuations.

The socSMCs concept also receives support from studies that highlight the role of active sensorimotor coordination for agent recognition in a simple virtual game involving perceptual crossing (Froese and Di Paolo, 2010; Auvray and Rohde, 2012; Froese et al., 2014; Lenay, 2017). In the experimental paradigm used by Auvray and Rohde (2012), two individuals move an avatar along a virtual line, on which they meet three kinds of objects: the avatar of the other player, the shadow of the other player, as well as a stationary object. While all objects feel the same (they produce a vibration) to the players, only one of them can feel and respond to co-presence: the other player’s avatar. This alone suffices for players to reliably identify one another in the virtual space, based on players’ ability to recognize mobile objects, as well as the fact that due to the interaction dynamic, they more frequently met their partner, versus their partner’s shadow.

Another line of work that generates insight into how social engagement emerges through interaction is provided by studies of musical improvisation. For instance, Walton et al. (2018) used a combination of interviews and behavioral modeling to better understand the interactions between pairs of jazz pianists. Their models relate musicians’ upper-body and musical movement (recordings of key-press timings and notes played) to changes in the musical environment (two different rhythmic background sounds), and the experience of successful and creative performance as inferred from analysis of the interviews. One of their main findings was that players’ experience was heavily influenced by how well they were able to co-create a narrative – a structure to guide their collaborative play and the emergence of new behaviors. Importantly, the study demonstrates a clear relation between the movement coordination of the players and the subjective experience of social engagement, thus supporting one of the predictions of the socSMCs concept.

A closely related field of research is the study of dyadic or group improvisation in the form of dance (Himberg et al., 2018; Kimmel et al., 2018). Akin the joint creation and negotiation of time in music, Himberg et al. (2018) focus on movement coordination (quantified by motion capture) and first-person appraisal thereof (inferred from interviews and questionnaires) as a vehicle for the aesthetic experience of togetherness, i.e., moments in which dancers experience heightened connection among the group, and a genuinely distributed sense of agency. The authors establish felt togetherness as a cross-sensory and inherently shared phenomenon that clearly relates to the agents’ coordination dynamics. Kimmel et al. (2018) provide a detailed phenomenological account, based on analysis of interview data, of how dancers co-create movement sequences in the explorative practice of contact improvisation. Constrained only by concerns for safety, collaboration and respect, dancers in contact improvisation deploy rolling, sliding, and falling movements to solve and create interactive challenges with their partner and the ground.

The relationship between social cohesion and interpersonal movement coordination is also revealed in experimental evidence from psychotherapeutic settings. For example, Ramseyer and Tschacher (2014) analyzed video-recorded therapy sessions and showed that both the amount of movement in patient and therapist, as well as the degree to which these movements correlate, positively predict therapeutic outcome (see also Tschacher et al., 2017; Moulder et al., 2018).

Another vast line of support for the intricate relations between bodily and personal or social dynamics comes from functional neuroanatomy. For example, the large body of work provided by Craig (2009a, b) provides detailed accounts of the neurophysiological overlap of brain regions and pathways associated with monitoring of bodily states, with areas and pathways implicated in emotion, one’s subjective experience of time, and other dimensions of social and self-awareness.

Together, these findings indicate that the skill to create and express oneself in coordinative structures in real-time, together with sensitivity to one’s own bodily sensations, contributes critically to the phenomenon of togetherness in social interaction dynamics. These studies support the proposal that a shared space of SMCs underlies agents’ experiences of an engaging social interaction, both in the sense of being safe and predictable, as well as inviting and stimulating.

## Conclusion

As discussed above, the socSMCs concept places joint action center stage and highlights in particular the situated and embodied sensorimotor processes that facilitate our participation in a shared social world. Our proposal, thus, extends action-oriented accounts of cognition (Varela et al., 1991; Clark, 1997; Noë, 2004; Engel et al., 2013b) to the interaction between different cognitive systems and broadens, in particular, the notion of SMCs beyond their application in the theory of individual cognition (O’Regan and Noë, 2001). In providing an overview of existing approaches to account for the complexity of dynamics present in human social cognition, we have attempted to show that novel approaches and perspectives emerge from this view of social interaction. However, key questions also remain open and need further investigation. This concerns, for instance, the exact nature of the grounding of subjective experiences of social engagement in the jointly maintained situated sensorimotor dynamics, as well as the translation of this insight into novel frameworks and interventions to support social interaction in both everyday life and clinical settings.

Pursuing the idea that SMCs may be applied in the context of social cognition, the central notion of our proposal is to ground social interaction in modes of sensorimotor and informational coupling, shifting the focus of study onto investigations of coordination dynamics as a vehicle of social entrainment. Our proposal shares aspects with interactionist concepts and joint action models of social cognition, but the socSMCs concept puts an even stronger focus on the role of low-level sensorimotor interaction dynamics for social entrainment and engagement. As we have discussed, this shift in emphasis has potential implications for the understanding of mechanisms underlying social cognition in the healthy brain but also in conditions of impaired social capabilities such as ASD. While work on the neural foundations of social cognition has, in the past decades, strongly focused on the capacity of the brain to mirror the actions of others, recent work suggests a key role for predictive mechanisms in social cognition in health and disease, and dynamic coupling between agents has become an issue of increasing interest in social neuroscience. In the context of ASD, modulation of social understanding through sensorimotor entrainment may even provide a new approach for augmentation of social capabilities. In a long-term application-oriented perspective, the socSMCs concept may also give rise to novel strategies for HRI and cooperation and may allow to introduce new concepts for robotics in training of social skills, in ambient assisted living, and caregiving.

## Author Contributions

AE, AM, and AL developed the core ideas of the concept discussed in this review. All authors contributed to writing of this article.

## Conflict of Interest

The authors declare that the research was conducted in the absence of any commercial or financial relationships that could be construed as a potential conflict of interest.

## Publisher’s Note

All claims expressed in this article are solely those of the authors and do not necessarily represent those of their affiliated organizations, or those of the publisher, the editors and the reviewers. Any product that may be evaluated in this article, or claim that may be made by its manufacturer, is not guaranteed or endorsed by the publisher.
